# Deep post-GWAS analysis identifies potential risk genes and risk variants for Alzheimer’s disease, providing new insights into its disease mechanisms

**DOI:** 10.1038/s41598-021-99352-3

**Published:** 2021-10-15

**Authors:** Zhen Wang, Quanwei Zhang, Jhih-Rong Lin, M. Reza Jabalameli, Joydeep Mitra, Nha Nguyen, Zhengdong D. Zhang

**Affiliations:** 1 College of Animal Sciences, Zhejiang University, Hangzhou, Zhejiang China; 2 Department of Genetics, Albert Einstein College of Medicine, Bronx, NY USA

**Keywords:** Computational biology and bioinformatics, Genetics, Neuroscience

## Abstract

Alzheimer’s disease (AD) is a genetically complex, multifactorial neurodegenerative disease. It affects more than 45 million people worldwide and currently remains untreatable. Although genome-wide association studies (GWAS) have identified many AD-associated common variants, only about 25 genes are currently known to affect the risk of developing AD, despite its highly polygenic nature. Moreover, the risk variants underlying GWAS AD-association signals remain unknown. Here, we describe a deep post-GWAS analysis of AD-associated variants, using an integrated computational framework for predicting both disease genes and their risk variants. We identified 342 putative AD risk genes in 203 risk regions spanning 502 AD-associated common variants. 246 AD risk genes have not been identified as AD risk genes by previous GWAS collected in GWAS catalogs, and 115 of 342 AD risk genes are outside the risk regions, likely under the regulation of transcriptional regulatory elements contained therein. Even more significantly, for 109 AD risk genes, we predicted 150 risk variants, of both coding and regulatory (in promoters or enhancers) types, and 85 (57%) of them are supported by functional annotation. In-depth functional analyses showed that AD risk genes were overrepresented in AD-related pathways or GO terms—e.g., the complement and coagulation cascade and phosphorylation and activation of immune response—and their expression was relatively enriched in microglia, endothelia, and pericytes of the human brain. We found nine AD risk genes—e.g., *IL1RAP*, *PMAIP1*, *LAMTOR4*—as predictors for the prognosis of AD survival and genes such as *ARL6IP5* with altered network connectivity between AD patients and normal individuals involved in AD progression. Our findings open new strategies for developing therapeutics targeting AD risk genes or risk variants to influence AD pathogenesis.

## Introduction

Alzheimer’s disease (AD) is a progressive, chronic neurodegenerative disorder with a long prodromal phase^1^. With a complex genetic etiology and a high heritability, estimated ranging from 60 to 80%^2^, AD is usually divided into two subgroups of diseases: the familial early-onset AD, caused by mutations in single genes including *APP*, *PSEN1*, and *PSEN2*, and the late-onset AD (LOAD), influenced by multiple common variants with low effect sizes^3^. Over the past decade, GWAS revealed a large number of AD-associated genetic loci (Supplementary Fig. S1 and Supplementary Table S1), including *SORL1*, *ABCA7*, *CLU*, *CR1*, *INPP5D*, *CD33*, *BIN1*, *PICALM*, *PTK2B*, and *APOE*, a locus that has been repeatedly validated across different studies^4^. Two recent meta-analyses of large cohorts of LOAD (*n* = 455,258 and 94,437) identified 29^5^ and 25^6^ risk loci, respectively. Interpretation of these results, however, remains elusive, because GWAS only detect statistical associations among a subset of all variants and ~ 86% of AD associated SNPs are non-coding (either intronic or intergenic, Supplementary Fig. S1). Studies have shown improvement on identifying potential risk AD genes by integrating GWAS and omics data. Most of those integration include rather limited or specific information (e.g. QTL or methylation data) alone to identify potential risk AD genes^7–9^. To better understand the biological mechanisms underlying AD etiology, the functional impact of genetic association signals needs to be extensively investigated to identify disease genes and risk variants underlying the genetic signals detected by GWAS.

To this end, we sought to integrate genomic data from multiple sources—e.g., GWAS signals from the GWAS Catalog, disease genes databases (MalaCards^10^, DISEASES^11^, and DisGeNET v5.0^12^), functional annotation of genetic variants (LINSIGHT^13^, ExPecto^14^, and PrimateAI^15^), and the 1000 Genomes Project—to predict AD risk genes and risk variants. In this study, we aimed to first compile a list of high-confidence AD risk genes derived from association signals, then systematically uncover the characteristics of the identified AD risk genes, including the level and variation of their expression in different types of cells, and finally use a computational framework that we developed to identify putative risk variants connected to AD risk genes. Our results provide novel biological insights into the genetic architecture, expression profiles, functional pathways involved in the AD etiology, and ultimately a basis for future therapeutic development for the disease.

## Results

### AD risk regions and risk genes

**Figure 1:**
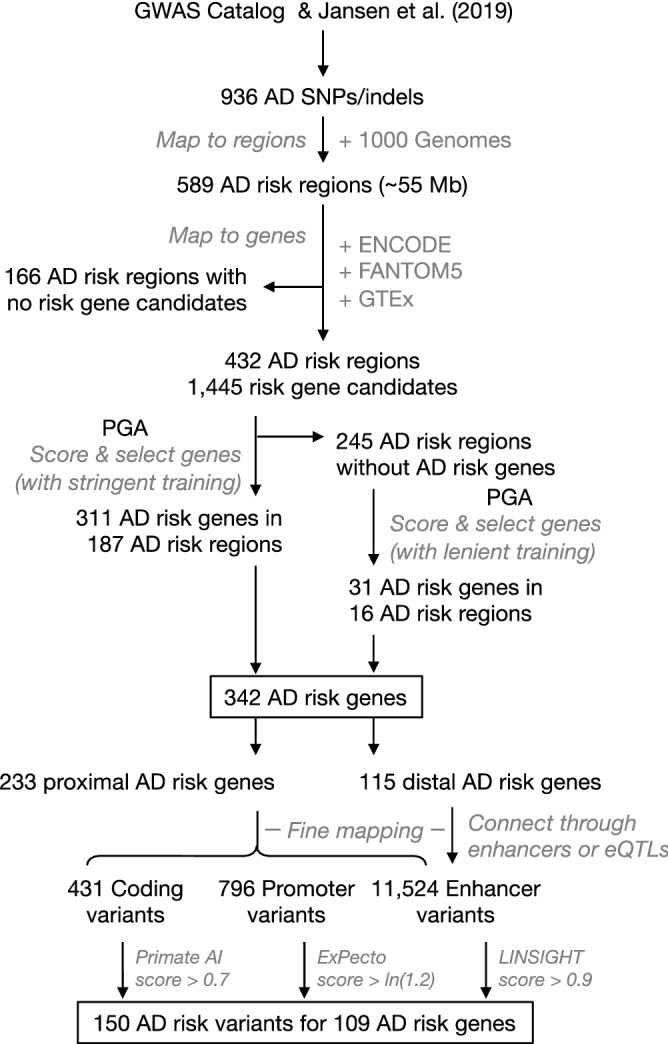
Flowchart of AD risk genes and risk variants prediction. Briefly, using 936 AD-associated signals and the 1000 Genome Project data, we first systematically identified genomic AD-risk regions, mapped them to genes (enhancer from ENCODE and FANTOM5 or eQTLs from GTEx were used to identity distal risk gene) to identify AD risk gene candidates, integrated gene network, annotation data and training gene sets to score all gene candidates for AD risk, finally selected genes with a score overpassed the threshold as AD risk genes. Next, all the variants located in the AD risk regions were grouped into coding, promoter and enhancer variants, and corresponding functional annotation of variants (PrimateAI, ExPecto and LINSIGHT) were used to prioritize these variants. The variants for identified AD risk genes with functional annotation score overpassed the threshold will be predicated as potential risk variants (see details in method).

**Figure 2:**
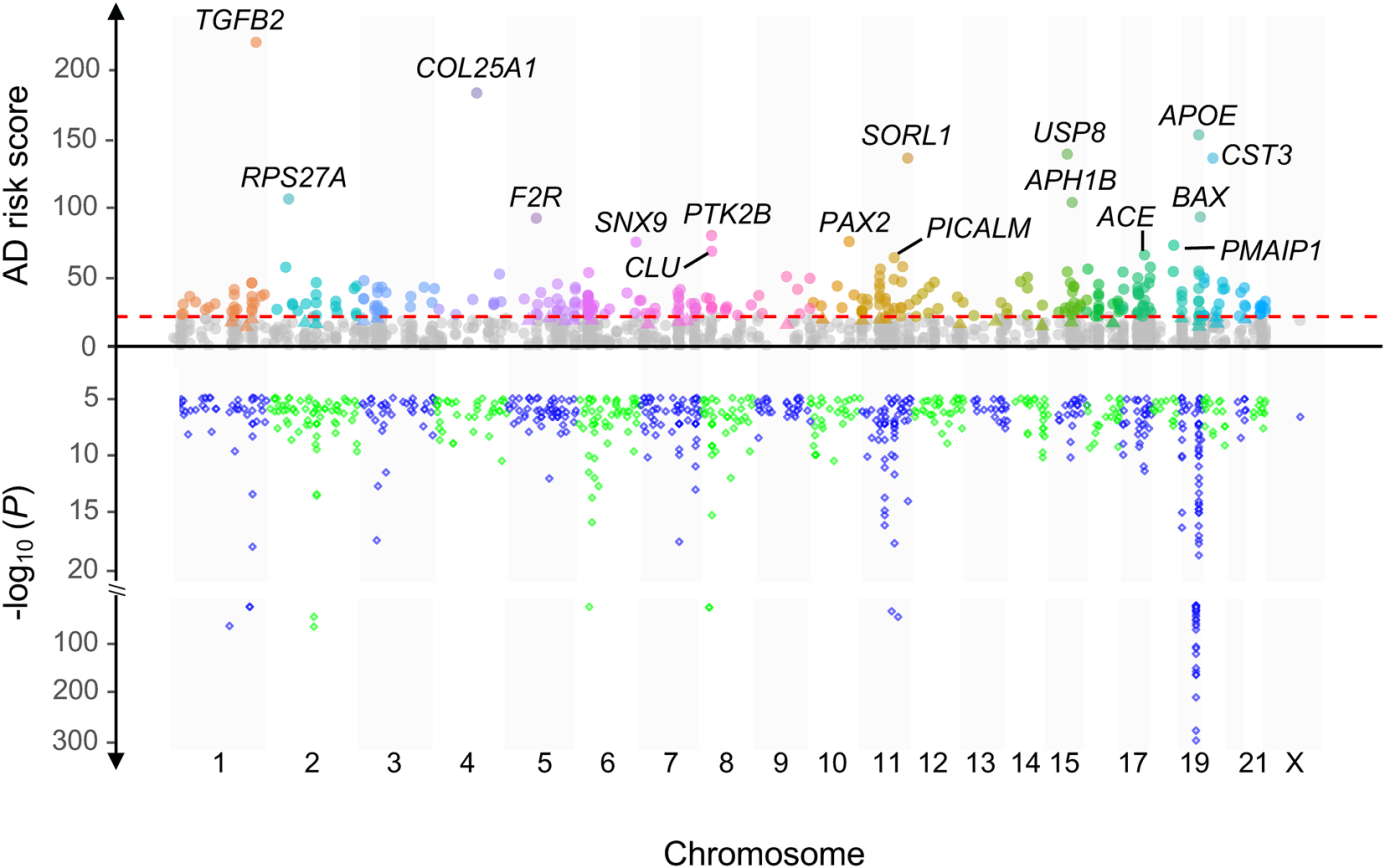
AD risk genes and AD-associated variants across chromosomes. The upper part of the figure shows the scores of AD risk gene candidates, which were calculated based on the stringent training gene set. Colored and gray dots represent AD risk gene candidates above and below the threshold (= 21.4, the red dashed line), respectively. The colored small triangles below the threshold represent additional AD risk genes predicted with the lenient gene training set. Top AD risk genes are labelled with their gene symbols. The upper part of the figure is the Manhattan plot of the 936 AD-associated SNPs that we collected from the GWAS Catalog and Jansen et al.^5^.

Using 936 GWAS AD SNPs and linkage disequilibrium, we identified 589 genomic risk regions, spanning ~ 55.0 Mb of the human genome (Fig. 1). Based on the genomic annotation of genes and regulatory information including enhancer and eQTL, we could connect 1,445 genes to 432 risk regions (Supplementary Fig. S2 and Supplementary Table S2). Among AD risk gene candidates, 967 are proximal genes, overlapping AD risk regions, and 506 are distal genes, linked to AD risk regions through long-range gene regulatory elements (e.g., enhancers) (Supplementary Table S2). 28 genes are both proximal for some risk regions and distal for other risk regions. 35 candidates were not scored due to their lack of GO annotation and/or exclusion from the gene functional linkage network. Among 1,410 scored candidates, 342 loci, distributed in 203 risk regions (Fig. 2 and Supplementary Table S3), surpassed the “high” threshold (see Method) and thus were considered as (putative) AD risk genes. They included 233 (68.1%) candidates proximal to AD risk regions, and additional 115 (33.6%) distal genes, which are likely to be regulated by regulatory elements in the risk regions (Fig. 1 and Supplementary Table S3). Comparing AD risk genes that we identified with ones reported by past GWAS, we found that 246 genes on our list are novel—they have not been identified as AD risk genes by AD GWAS so far, and many of on our list such as *CR1*^16^, *ABCA*7^17^, *TREM2*^18^, *SORL1*^19^ and *BIN1*^20^ have been reported to play import roles in the pathologies of AD.

### KEGG pathways and GO biological processes enriched with AD risk genes

**Figure 3:**
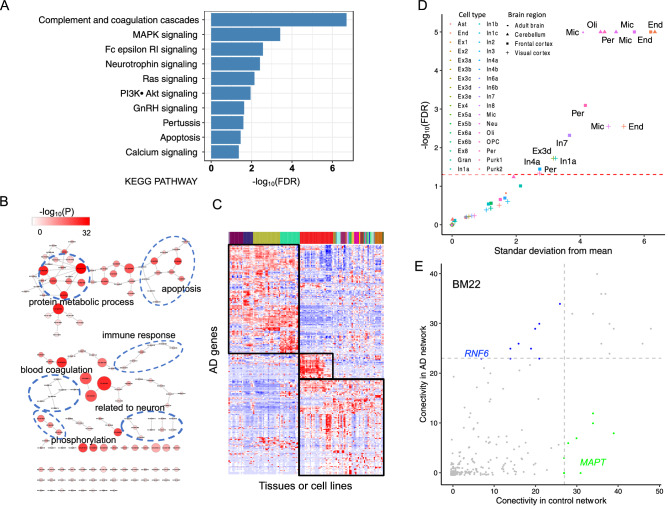
Functional annotation of AD risk genes. (**A**) KEGG pathways and (**B**) GO terms of biological processes enrichment of AD risk genes. In (**B**), each dot represents a significantly enriched GO term, whose –log_10_(*P*-value) and AD risk gene count are indicated by the color and size of the dot, respectively. (**C**) Expression of AD risk genes in different tissues. Three expression clusters are indicated by the black boxes. See Supplementary Fig. S3 for details. (**D**) Cell type enrichment analysis. We analyzed the expression of AD risk genes in different types of single cells from frontal and visual cortices, cerebellum, and adult brain. The red dashed line represents FDR = 0.05, and cell types whose transcriptomes were significantly enriched with the expression of AD risk genes are shown, with their brain regions indicated. Used cell type abbreviations: Ast, astrocytes; End, endothelial; Ex, excitatory; Gran, granule; In, inhibitory; Mic, microglia; Neu, neuron; Oil, oligodendrocytes; OPC, oligodendrocytes precursor; Per, pericytes; Purk, purkinje. (**E**) Network connectivity of AD risk genes in the co-expression network of both AD patients and normal controls in the brain region BM22. Each dot represents an AD risk gene. The gray dashed line marks the threshold for network hub genes either in the co-expression network of AD patients or normal controls. Blue and green dots represent network hub genes only in the co-expression network of AD patients and only in the co-expression network of normal controls, respectively. See Supplementary Fig. S6 for details and other brain regions.

Functional enrichment analysis of 342 AD risk genes using DAVID^21^ showed that AD risk genes were overrepresented in 10 KEGG pathways (FDR < 0.05) and with 151 GO terms (adjusted *P* < 0.01 after the Bonferroni correction) (Fig. 3A,B and Supplementary Table S4), many of which are highly relevant to the AD pathology.

### Clustered expression of AD risk genes in different human tissues

Based on their expression profiles in different human tissues, AD risk genes can be clustered into three groups (Fig. 3C and Supplementary Fig. S3). The first group of 32 genes was expressed almost exclusively in the central nervous system (CNS), especially the frontal and the prefrontal cortices. Many genes in this group, such as *BIN1*, *MAPT*, and *CNTNAP2*, have been implicated in the pathogenesis of AD^22–24^. The second group included 131 genes actively expressed in the immune cells such as B and T lymphocytes. 115 genes in the third group were expressed across a wide range of different tissues, including the CNS. Many genes in this group, such as *APOE*, *CR1*, and *EPHA1*, are known to be associated with AD. Human studies clearly indicate that ApoE isoforms differentially affect Aβ aggregation and clearance^4^, and *CR1* may play a role in the clearance of Aβ^25^.

### High expression of AD risk genes in microglia, endothelia, and pericytes of human brain

The expression of AD risk genes was significantly enriched in microglia, endothelia and pericytes in the frontal and the visual cortices and cerebellum from human adults (Fig. 3D and Supplementary Table S5). The high-expression profile was evident for many genes (Supplementary Fig. S4), which were enriched for microglial markers (e.g., *HLA-DRA* and *TREM2*), endothelial markers (e.g., *CD34*), and pericyte markers (e.g., *NR1H3*).

### Connectivity of AD risk genes in co-expression network

We carried out the gene co-expression network analysis across four brain regions to examine the gene regulation patterns among AD risk genes. Overall, we observed that connections among AD risk genes are less (BM10: *P* value = 0.50; BM22: *P* value = 5.8E-3; BM36: *P* value = 0.01; BM44: *P* value = 8.6E-7; all two-sided t-tests) in AD patients compared to normal controls (Supplementary Figs. S5 and S6). For each brain region, we considered top 20 genes with most interactions with other genes as network hubs. We found that hub genes with high connectivity in AD patients—e.g., *ARL6IP5* (BM10), *RNF6* (BM22), *TP53INP* (BM36), and *GGH* (BM44) —tended to have low connectivity in healthy individuals (Fig. 3E and Supplementary Fig. S5). On the other hand, many hub genes in normal people—e.g., *LMTK2* (BM10), *SPPL2A* (BM22), *MAPT* (BM44), and *USP8* (BM36)—usually had low connectivity in AD patients.

We also analyzed co-expression of AD risk genes at the proteomic level and observed similar patterns that the AD risk genes were less connected among AD patients than normal controls in the ACG region (*P* value = 2.2E-16, two-sided t-test), while opposite pattern in the FC region (*P* value = 1.6E-3, two-sided t-test, Supplementary Fig. S7). For example, *ARL6IP5* and *GGH* were network hubs in AD patients but less connected in controls in the FC region (Supplementary Fig. S8). In the ACG region, we observed network hubs such as *PTK2B*, *SPARC*, and *RAD50* showing large alteration between AD patients and controls (Supplementary Fig. S8). SPARC is a matricellular protein which can facilitate the migration of immune cells (e.g., blood-derived dendritic cells). Although its role in AD-related neuroinflammation is still not clear, a study has shown that there are significant alterations in its expression and it collocates to Aβ protein deposits in AD brain tissues^26^.

### Expression of AD risk genes in human brain and its connection to disease survival

Using data from three studies of differential gene expression between AD cases and controls in different brain regions^27–29^, we found 171 (50%) AD risk genes were differentially expressed in at least one brain region, including 102 up-regulated genes, 64 down-regulated genes, and 5 genes showing both up- and down-regulation in different brain regions (Supplementary Table S6). Differential expression of AD risk genes was either widespread, occurring in multiple brain regions, or limited to a specific brain region. *TGFB2,* the highest ranked risk gene, was up-regulated in frontal cortex (FC), central nervous system (CNS), temporal cortex (TCX), superior temporal gyrus (STG) and parahippocampal gyrus (PHG), while *PTK2B* was down-regulated in brain cerebellum (CBE), TCX, and PHG. *COL25A1*, the second highest ranked risk gene, and *PMAIP1* were separately down- and up-regulated only in the TCX region. Differential expression of some AD risk genes was discordant in different brain regions. For example, *ApoE* and *CST3* in AD patients were up-regulated in TCX region but down-regulated in cerebellum.

Since AD is mainly a late-onset neurodegenerative disorder, we examined how AD risk genes are expressed specifically among adults. Using a binarization procedure^30^, we analyzed their spatiotemporal expression patterns using RNA-seq data from BrianSpan. Although no strong pattern was found (Supplementary Fig. S9), the proportion of AD risk genes with dramatically suppressed expression was increased at the age of 40 compared to early ages. The proportion of AD risk genes that tend to be transcriptionally actively was relatively higher at the early ages (of 23 and 30) in comparison to the old ages (of 36 and 40). We next examined the spatiotemporal expression pattern of AD risk genes during the development of the frontal cortex across an extended range of ages (from 18.05 to 78.23). We did not observe any distinct expression pattern across this range of ages.

**Figure 4:**
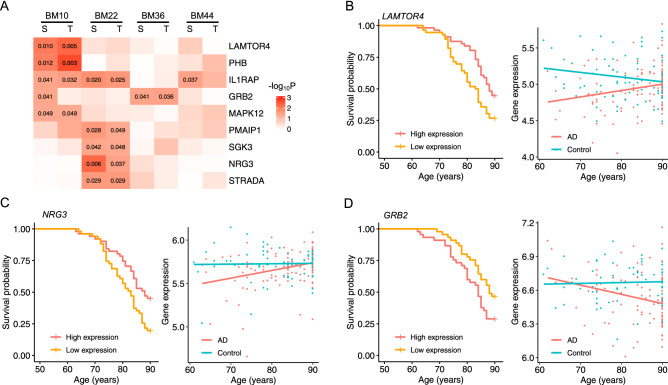
AD survival curves and gene expression trajectories with age. We analyzed the expression of each AD risk gene to see how it may affect the survival of AD patients with low and high expression levels and how it trends with age in AD patients and normal controls. (**A**) The heatmap of *P*-values of Kaplan–Meier survival analyses and gene expression trend tests across four brain regions (see Methods). Nine AD risk genes with at least one *P*-value less than 0.05 (labeled) are included in the heatmap. This figure includes AD survival curves and gene expression trajectories with age for three genes with *P* < 0.05: (**B**) *LAMTOR4* in the brain region BM10, (**C**) *NRG3* in the brain region BM22, and (**D**) *GRB2* in the brain region BM36. See Supplementary Fig. S10 for plots of other significant results.

As a chronic neurodegenerative disease, AD starts slowly and gradually worsens overtime. We hypothesized that genes whose expression correlates with AD progression may mark AD severity and thus can be used to predict AD prognosis. To test this hypothesis, we assessed the impact of AD genes on survival using the Kaplan–Meier analysis. Based on expression levels, nine genes—*NRG3*, *IL1RAP*, *PMAIP1*, *STRADA*, *SGK3*, *LAMTOR4*, *MAPK12*, *PHB*, and *GRB2*—separated AD patients into low- and high-risk groups with different disease survival (*P* < 0.05). Their expression also trends differently with age between healthy individuals and AD patients in at least one brain region (*P* < 0.05) (Fig. 4 and Supplementary Fig. S10).

### Predicted AD risk variants

Using the computational framework that we developed for this project, we predicted 150 unique potential risk variants (51% of them located in risk regions with p-value < 5E-8) for 109 AD risk genes (Supplementary Table S7 and Fig. S11A). To evaluate this prediction, we analyzed their effect on sequence motifs of transcription factors binding sites (TFBS) and compared these to the eQTL data from GTEx. Motif analysis revealed that 69 predicted risk variants (46%, Supplementary Table S7) cause either gain or loss of TFBS motifs, likely affecting TF binding. Among them, 32 (21%, Supplementary Table S7) have also been identified as eQTLs. Together, 85 (57%) of the predicted risk variants can be functionally annotated (Supplementary Fig. S11B). Three modules were developed in our computational framework to predict risk variants in different functional genomic regions:

#### Coding variants

**Figure 5:**
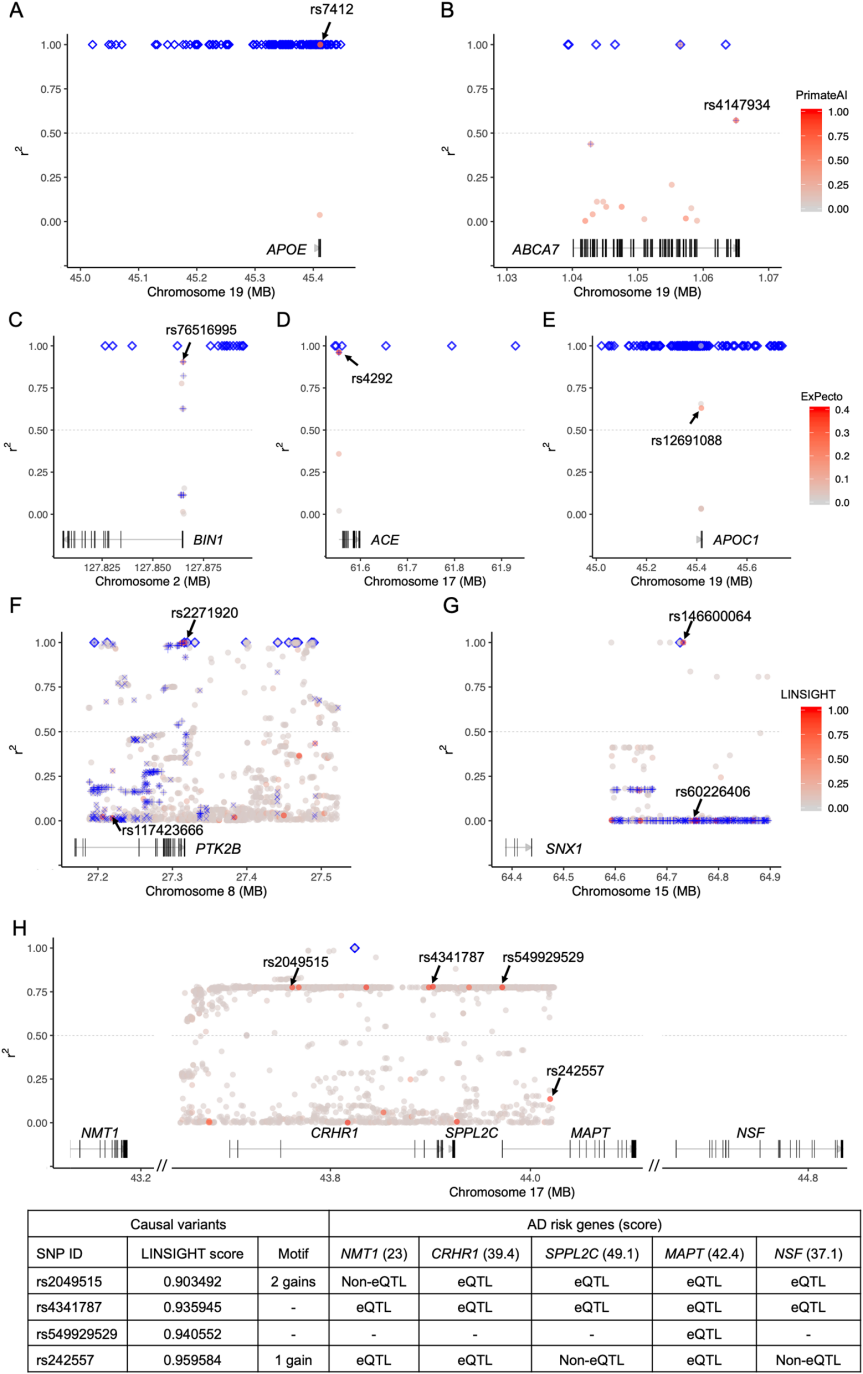
Predicted risk variants. Plotted are examples of coding, promoter, and enhancer risk variants identified using PrimateAI (**A**, **B**), ExPecto (**C**–**E**), and LINSIGHT (**F**–**H**), respectively, with their affected AD risk genes. Each dot represents a variant, whose annotation score is indicated by the color of the dot. Predicted risk variants are marked by arrows with their SNP IDs. Blue diamonds represent AD-associated variants identified by GWAS. Plus and cross signs represent eQTLs and variants located in enhancers. The gray dashed line represents LD *r*^2^ = 0.5.

We predicted 54 risk coding variants. For example, rs7412 and rs4147934 are two missense coding SNPs, each in high LD (*r*^2^ > 0.5) with one of the AD GWAS lead SNPs in its corresponding risk region, were predicted as risk variants (Fig. 5 A and B, Supplementary Table S7). rs7412, in *APOE* with a PrimateAI score = 0.80*,* is a well-known variant reported to be associated with AD. rs4147934, in *ABCA7* with a PrimateAI score = 0.78, has been proposed as a functional candidate variant accounting for the GWAS signal at *ABCA7* locus in Caucasians^31^. Although AD risk from rs4147934 is probably population-specific since its association signal was not replicated in the African American cohort^32^, our analysis provides additional evidence in support of its causal role in AD and thus its impact in non-European ancestry populations merits further investigation.

#### Non-coding variants in promoters

We also predicted 33 risk promotor variants in brain tissues or cells (Fig. 5C–E), including rs76516995 (ExPecto score = 0.194) for *BIN1* in astrocytes, rs4292 (ExPecto score = 0.390) for *ACE* in neural cells, and rs12691088 (ExPecto score = 0.224) for *APOC1* in astrocytes. rs76516995 and rs4292 have also been identified as eQTLs by GTEx, while rs12691088 has been shown to be associated with AD-related phenotypes in multiple brain regions^33^.

#### Non-coding variants in enhancers

We predicted 64 risk enhancer variants. Two of them, rs2271920 (LINSIGHT score = 0.951) and rs117423666 (LINSIGHT score = 0.966) Fig. 5F and Supplementary Table S7), are risk variants for risk gene *PTK2B.* rs2271920 is an AD GWAS lead SNP itself^34^. As a GTEx eQTL, it changes the expression of *PTK2B*, likely by altering the binding sites motif of BCL6 and ZNF467. For the *SNX1* locus, we also predicted two risk enhancer variants: rs146600064 (LINSIGHT score = 0.914) and rs60226406 (LINSIGHT score = 0.977) (Fig. 5G and Supplementary Table S7). rs146600064 is in total LD (*r*^2^ = 1) with the lead AD SNP rs74615166^3^. In another risk region indexed by the AD lead SNP rs7207400^34^, several predicted risk variants—rs2049515, rs4341787, rs549929529, and rs242557—are connected to multiple AD-risk genes—*NMT1, CRHR1, SPPL2C, MAPT*, and *NSF* (Fig. 5H and Supplementary Table S7). Although a disease risk gene could be influenced by multiple risk variants, we examined additional data—e.g., TFBS motifs, eQTL, and ClinVar—to further prioritize them. rs549929529 is most likely a risk variant for *MAPT* as it is reported in ClinVar to be associated with 'MAPT-Related_Spectrum_Disorders'. Compared with other SNPs, rs4341787 is in the highest LD (*r*^2^ = 0.779) with the AD lead SNP rs7207400 and is an eQTL identified by GTEx for *NMT1*. Also, in high LD (*r*^2^ = 0.775) with rs7207400, rs2049515 could be a risk variant for multiple genes as it is an eQTL for *CRHR1*, *SPPL2C*, and *NSF* (Fig. 5H and Supplementary Table S7). Moreover, rs1522388, a predicted risk variant (LINSIGHT score = 0.979) for *FLNB,* was also identified as a reporter assay QTL in HepG2 cell line^35^, which experimentally demonstrated its functional impact as a regulatory variant.

To date, the functional impact of the aforementioned variants is still poorly understood in AD etiology, but our findings provide promising risk variant candidates for further experimental validation, which will in turn identify potential drug targets for the development of AD treatment. To explore the molecular function of the predicted risk variants, we examined eQTLs (FDR < 0.05) identified in three brain regions (temporal cortex, dorsolateral prefrontal cortex, and cerebellum) in an AD cohort (Synapse: syn17015233^36^). We found 16 (11%, Supplementary Table S8) risk variants were AD-related eQTLs. Several risk variants—e.g., rs2236393 for *CDH3* and rs12752439 for *HSPG2*—were not identified as eQTLs by GTEx but instead are AD-related eQTLs. This analysis provides direct evidence for their involvement in AD pathogenesis.

## Discussions

GWAS have uncovered thousands of genetic variants that influence risk for complex human diseases. However, there is still a large gap between the statistical associations linking locus and trait and the functional impact of risk variants underlying disease risk. Multiple factors have made it difficult to bridge this gap. First, the association of a locus with disease does not reveal the underlying causal variant as many co-inherited variants in strong LD with one another at the locus often have statistically indistinguishable disease associations. Second, genes affected by the causal variants are usually unknown. Consequently, functional studies aimed at determining the causal genetic variants and the biological mechanisms underlying the observed disease association have lagged. Thus, it is important to not only identify disease risk genes but also explore their risk variants. Responding to this need, we proposed a post-GWAS computational framework that could predict the risk variants for a specific disease.

Using this framework for AD, we predicted 342 AD risk genes and, for 109 of them, 150 risk variants. The replication of finding many well-known AD risk genes using our framework shows a reliability of our approach. For example, the predicted AD risk genes with the highest risk scores are well-known AD risk genes, such as *ApoE* and *SORL1* have already been shown to functionally underlie the disease pathology^37^ (Fig. 2). Moreover, we also provide AD risk genes with highest risk scores as candidates, such as *TGFB2*, *CST3* and *USP8* genes, for researchers to further study their functions in AD. The role of the *TGFB2* gene in AD is still poorly understood, although it was found to autocrinally induce the apoptosis of primary cortical neurons^38^ and up-regulated in multiple brain regions among AD patients. TGFβ signaling and *COL25A1* play important roles in the pathogenesis of AD^39,40^, while *CST3* may offer neuroprotection against AD^41^. *USP8* gene depletion leads to decreased levels and activity of *BACE1*, the rate-limiting enzyme in the production of amyloid-β^42^.

Several recent studies explored the connection between diseases and genetic variants in coding sequences^15^, non-coding regions^13^, and promoters^14^ in human genome. Our framework for post-GWAS analysis integrates these data to predict disease causal variants. Over 90% of disease-associated variants found in GWAS are located in non-coding regions, and aggregate analysis of them has shown that they are significantly enriched in transcription-factor-occupied regions and DNase I hypersensitive sites, most of which overlap enhancer regions^43^, suggesting that many disease loci identified by GWASs influence transcriptional output of one or more target genes through enhancer variants. However, it has been difficult, computationally or experimentally, to identify risk variants in enhancers for any disease. In our study, AD risk variants located in promoters or enhancers likely modulate the disease risk by influencing the expression of 85 AD risk genes, among which 50 (58.8%) are differentially expressed in at least one brain region, including 27 up-regulated genes, 20 down-regulated genes, and 3 genes showing both up- and down-regulation in different brain regions (Supplementary Tables S6 and S7). This indicates that these genes are likely to modulate the disease risk through altering gene expression in brain regions. For AD, we predicted 62 risk enhancer variants. For over 66% of them, there is additional support for their functional impact on enhancer: 16 are known eQTLs and 29 can alter transcriptional factor binding through gain or loss of TFBS motifs, implying a strong reliability of these variants should be the risk variants.

AD risk genes enriched in KEGG pathways and GO biological processes highly relevant to the disease pathology. The most significantly enriched KEGG pathway is the complement and coagulation cascades. Increasing evidence suggests that deregulation of the complement cascade is a contributing factor leading to chronic inflammation and neurodegeneration observed in AD^44,45^. The complement system plays an important role in the innate and adaptive immune responses, restricts amyloid plaque formation, and helps clearance of plaque components associated with AD^46^. AD risk genes are also enriched in MAPK signaling pathway, which contributes to the AD pathogenesis through multiple mechanisms, including the regulation of neuronal apoptosis and phosphorylation of APP and tau^47^. Additional enriched pathways, such as GnRH signaling, PI3K-Akt signaling, neurotrophin signaling, and calcium signaling, all have been shown to likely play a role in AD^48–51^, whereas Ras signaling may play an important role in aging^52^. Many enriched GO terms—e.g., “nervous system development”, "neurogenesis”, “neuron differentiation”, “neuron development”—are related to the development and differentiation of neurons. Some are directly related to AD: e.g., “phosphorylation (*P* < 0.01)”, “activation of immune response (*P* < 0.01)”, and “learning memory” are part of the currently predominant hypothesis of the pathogenesis of AD^53,54^. In additional, several blood-related GO terms are enriched: “blood vessel development”, “vasculature development”, “negative regulation of blood coagulation”, and “hemopoiesis”. Increasing evidences has shown that the hematopoietic system may contribute to the initiation and/or progression of AD^55^.

Clustered expression of our AD risk genes in the second group actively expressed in the immune cells such as B and T lymphocytes, implying immune related genes might highly correlate with AD. Recent studies showed that inflammation contributes to the pathogenesis of AD^56^. It is important to note that many immune related genes, such as *TREM2*, *INPP5D*, *CD34,* and *CD55*, were included in this group. TREM2 is a cell surface receptor of the immunoglobulin superfamily protein expressed in microglia in the CNS. As a potential key molecule in AD pathogenesis, it might protect against neurodegeneration by promoting phagocytosis to clear apoptotic neurons^57^ and a broad array of microglial functions in response to Aβ deposition^58^. INPP5D plays a significant role in inflammatory responses and has been implicated in the pathogenesis of late-onset AD through the regulation of microglial and myeloid cell function^3^. This suggests that immune processes may directly contribute to the pathology and progression of AD, rather than being the consequence of the neurodegeneration. Cholesterol metabolism-associated genes in this group, such as *SORL1* and *ABCA7*, have been linked to AD in previous studies. Studies showed that the suppression of *SORL1* expression contribute to the overexpression of Aβ and an increased risk of AD^59^. *ABCA7* is a genetic risk factor for late-onset AD and may participate in the regulation of Aβ homoeostasis in the brain^60^. Moreover, this group also includes tauopathy-associated AD risk genes, such as, *PTK2B* and *PICALM*. Previous studies in *Drosophila* indicated that *PTK2B* acts as an early marker and in vivo modulator of tau toxicity^61^. Cell-based and in vivo data showed that perturbations of *PICALM* levels might be a key for the regulation of autophagy and tau levels and therefore essential for modulating tau toxicity^62^.

AD risk genes are expressed in many different types of brain cells. We found that they are over-expressed in microglia, endothelia, and pericytes from three different regions of adult brains, consistent with previous reports^63,64^ showing that these cell types are likely to be associated with AD pathology. Previous studies showed that microglia are the primary cells contributing to the initiation of the immune response to AD pathology, and the aberrant microglial activation is a causal factor for the development of AD^65^. Recent studies also suggests endothelial dysfunction may be involved in the pathogenesis of AD^66^. Pericytes, cells in the blood–brain barrier, degenerate in AD and are reported to control multiple steps of AD-alike neurodegeneration cascade in mice overexpressing Aβ-precursor protein^67^. Moreover, oligodendrocytes are only significantly enriched with overexpressed AD risk genes in cerebellum. The major function of oligodendrocytes is the formation of myelin, whose breakdown is associated with AD^68^. We also observed over-expression of AD risk genes in specific cell types or brain regions or both. For example, only oligodendrocytes from cerebellum showed active expression of AD risk genes. When frontal cortex with visual cortex were compared, only In4a and In7 cells from the former and In1a cells from the later showed enriched expression for AD risk genes. These findings were replicated using a single-cell transcriptome data set^63^ (Fig. 3D). A previous expression weighted cell-type enrichment analysis of a set of 178 AD risk genes using the same data set failed to identify significantly enriched cell types^64^, likely due to the incomplete list AD risk genes used in that analysis.

AD is a progressive neurodegenerative disease that involves alteration of gene expression at the whole transcriptome level. The perturbation in the sub-networks of co-expression involving AD risk genes can partially reflect AD progression. Finding the altered network hub genes involved in AD progression may help identify AD biomarkers.

We found hub genes, such as *RNF6*, *TP53INP*, and *GGH* genes, with high connectivity in AD patients but tended to have low connectivity in healthy individuals. Rnf6, a ring-finger-dependent ubiquitin ligase, functions for proteasomal degradation in axonal growth cones of primary hippocampal neurons in mice by regulating the levels of *Limk1*, which play a crucial role in neurodevelopment and synaptic plasticity^69^. *TP53INP1*, a major regulator of p53 in response to oxidative stress^70^, is a tumor suppressor associated with malignant tumor metastasis in breast, liver, pancreas, and stomach and plays a critical role in cancer progression. Interestingly, previous studies showed inverse correlation between cancer and AD^71^. It has been reported that tripeptide GGH might be used for Cu chelation therapy for AD treatment as Cu ion level was reported to be elevated in AD brains and accumulation of amyloid plaques leading to metal homeostasis dysregulation^72^. Also, we found many hub genes, such as *LMTK2*, *MAPT*, *USP8*, and *SPPL2A* genes, in normal people turn into have low connectivity in AD patients. *LMTK2* may contribute to the neurodegenerative process by disrupting axonal transport, tau hyperphosphorylation and enhancing apoptosis^73^. Its expression is decreased in a tau mouse model of AD^74^. As one of the deubiquitinases, which play a critical role in regulating synaptic function and whose dysfunction results in several neurological disorders, *USP8* has been shown to be associated with AD^42^, Parkinson’s disease, and Lewy body disease. *MAPT* encodes tau protein, whose hyper-phosphorylation and subsequent intracellular neurofibrillary entanglement is one of definitive neuropathological hallmarks of AD. SPPL2a is an intramembrane protease of lysosomes/late endosomes and plays a critical role in regulation of intramembrane proteolysis in B cells and the regulation of innate and adaptive immunity^75^.

As expected, many predicted AD risk genes were also identified by survival analysis as predictors for AD prognosis. They include *IL1RAP*, *PMAIP1*, *LAMTOR4*, and *GRB2*. IL1RAP, a key immune signaling factor, impacts amyloid accumulation by modulating the activity of microglia and is crucial in clearing brain amyloid and limiting plaque growth^76^. PMAIP1 is an essential mediator of p53-dependent apoptosis, an important biological process in neurodegenerative disorders^77^. LAMTOR4 is a component of the Rag-Regulator complex and an essential regulator of lysosomes in microglia. Its absence has been shown to result in diminished number of microglia in Zebrafish^78^. GRB2, a cytoplasmic protein, are involved in protecting the cytoskeletal architecture in AD-like conditions^79^ and interacts with the C-terminal fragment or tyrosine-phosphorylated APP. This interaction intensifies significantly in neuronal cells and AD brains^80^. Finally, it is worth noting that the predictor genes for AD prognosis are region-specific, as none was identified in BM44 and only one (*GRB2*) in BM33. Because brain samples can only be obtained after death, with the assumption that postmortem data are indicative of the long-term gene expression state before death, we performed the survival analysis to detect predictors for AD prognosis and to understand the expression patterns of AD risk genes. Although this assumption may not be true for every gene and thus could lead to bias in our results, the survival analysis can nevertheless be helpful in detecting potential predictors for AD prognosis, which can be experimentally validated and examined.

Limitations. In this study, we developed an integrated computational framework aimed to predict both disease genes and corresponding risk variants using multiple omics data sets. Although our results showed that the predicted risk variants are likely to be functional, they need be experimentally validated and studied using transgenic AD mouse models^81^ and the luciferase reporter assay technology. Moreover, this study did not consider the impact of other critical covariates such as sex and APOE on the results.

Overall, in this study we prioritized new potential AD risk genes and risk variants from AD GWAS by integrating comprehensive omics data. Moreover, we provided comprehensive functional annotation to those putative risk genes and variants. Our findings will facilitate translating AD genetics to potential therapeutic target genes/variants and can be used to devise new strategies for AD therapeutics development.

## Materials and methods

In this study, we developed a computational pipeline (Fig. 1) that integrates multiple data sets to predict AD risk genes as well as their risk variants, and systematically investigated their characteristics and functions. We first used the PGA software tool^82^ to identify AD risk genes on the whole-genome level, and then examined their expression patterns across different human cells and tissues and during brain development, their co-expression network, and survival curves at the transcriptomic level. Next, using an analytical framework that we developed, we predicted potential risk variants for identified AD risk genes.

### Data sets and their sources

*AD GWAS SNPs*. From 58 AD GWAS collected in the GWAS Catalog^83^ (as of December 2018) and the latest study^5^ (meta-analysis of AD-proxy samples and LOAD samples, a valuable resource for our study to identify potential AD risk regions and is beneficial for our study to identify potential AD risk genes and variants), we collected 936 AD-associated SNPs (*P* < 1E−5, Fig. 2 and Supplementary Table S1) with a lenient *P* value (suggestive evidence of association^84^) to contain more potential AD risk genes candidates being analyzed.

#### AD genes for PGA training

338 AD genes (Supplementary Table S9) were collected from three disease-gene databases: MalaCards^10^, DISEASES^11^, and DisGeNET v5.0^12^. They were separated into two groups: a stringent set of 98 AD genes supported by at least two of these three disease-gene databases and a lenient set of 240 AD genes supported by only one of the three databases for AD.

#### Differentially expressed genes (DEGs)

We assembled a list of 10,314 genes that have been shown in three studies^27–29^ to be differentially expressed in nine brain regions between AD patients and normal controls (Supplementary Table S10).

#### Variant and genome annotation

We used multiple annotation metrics for various data analyses in this study, including scores from LINSIGHT^13^, ExPecto^14^, and PrimateAI^15^, enhancer-gene connection from HEDD^85^, eQTL from GTEx v.6.p^86^, and Synapse (syn17015233^36^).

### Identification of genomic risk regions and putative candidate genes

Using PGA^82^, able to integrate different types of data to uncover plausible risk genes implicated by GWAS signals that might be missed by other methods, we first systematically identified genomic AD-risk regions. Each AD risk region is an LD block that is seeded with a GWAS-identified AD variant and defined by 1000 Genomes Project variants that are in substantial LD (*r*^2^ > 0.5^30^) with the AD variant and less than 400 kb away from it. Overlapping or close (< 250 kb) LD blocks were merged. AD risk gene candidates consisted of proximal genes that overlap (after extending their ranges by 20 kb on each end^87^) these genomic AD risk regions and distal genes that are more than 20 kb away from AD risk regions but are linked to them by regulatory elements within them. We then integrated both gene network and annotation data with GWAS signals to score all candidates for AD risk. Gene regulatory information about enhancers and eQTLs in 44 human tissues (including non-brain tissues, which could provide complementary gene regulatory information) and their target genes from HEDD^85^ and GTEx (v6p)^86^ were used to identify distal AD risk gene candidates. Risk gene candidates were scored by:

$$ S_{g} = \alpha S_{f}^{\left( n \right)} + \left( {1 - \alpha } \right)S_{f}^{\left( a \right)} (0 < \alpha < 1) $$

in which $S_{g}$ is gene score, $S_{f}^{\left( n \right)}$ and $S_{f}^{\left( a \right)}$ are the network and annotation-based scores, respectively, and α is a coefficient controlling the relative weights of these two scores on the final gene score (see more details in “scoring risk gene candidates” section in^82^) with aforementioned training gene sets, and high-scoring candidates with scores greater than the threshold (achieves a prediction precision ≥ 0.8) estimated with either the stringent (= 21.4) or the lenient (= 13.1) gene set were taken as (putative) AD risk genes for downstream analysis.

### Characterization of AD risk genes

#### Functional enrichment analysis

We used DAVID^21^ to identify KEGG^88^ pathways and GO terms^89^ enriched with AD risk genes. *P*-values were adjusted for multiple tests using the Bonferroni or FDR methods.

#### Expression weighted cell-type enrichment (EWCE) analysis

We used EWCE (v1.2.0)^90^ to identify brain cell types, which are more likely to be affected by AD risk genes. Single-cell RNA-seq data from 33 types of cells (Supplementary Table S5) from human adult brains (20–51 years old; a total of 35,289 cells; from the visual cortex, frontal cortex, and cerebellum)^91^ were used in this analysis. The average expression across all samples for each cell type was used to determine enriched expression by EWCE.

#### Tissue gene expression analysis

To investigate gene-tissue expression specificity, we examined the expression profiles of AD risk genes across different tissues from the Gene Enrichment Profiler (http://xavierlab2.mgh.harvard.edu/EnrichmentProfiler/help.html)^92^, which catalogs normalized expression values of ~ 12,000 genes across 126 primary human tissues. We grouped the putative risk genes into different clusters according to their different expression profile across tissues using the Euclidean distance and the Ward’s clustering method^93^. The heatmap of the gene expression was plotted using R ‘gplots’ package with the ‘heatmap.2’ function.

#### Analysis of expression of AD risk genes during human brain development

Temporal gene expression patterns in different brain tissues and their regulation across the lifespan reveal molecular mechanisms involved in the formation, maturation, and degeneration of the human brain. Here, we examined the spatiotemporal expression patterns of AD risk genes in adult human brain. We first compiled gene expression profiles across ten regions of adult human brains from individuals of 19 to 40 years old, using RNA-seq data from BrainSpan^94^ (http://www.brainspan.org/, as of April 2018). We then explored the temporal dynamics of gene expression in a more specific human brain region (i.e., prefrontal cortex) during brain development from fetuses to older adults, using spotted oligonucleotide microarrays yielding data from Brain Cloud^95^ (http://braincloud.jhmi.edu/, as of May 2018).

#### Gene co-expression networks

We built gene co-expression networks using transcriptomic and proteomic data. From Synapse (syn7391833^96^), we collected expression profiles of ~ 23,200 genes (included 315 AD risk genes) in four human brain regions from normal controls and AD patients with the definitive disease status: frontal pole (Brodmann area 10, BM10) with 111 cases and 76 controls, superior temporal gyrus (BM22) with 102 cases and 65 controls, parahippocampal gyrus (BM36) with 90 cases and 68 controls, and inferior frontal gyrus (BM44) with 90 cases and 64 controls. From Synapse (syn10239444^97^), we also collected proteomic profiles of ~ 10,000 gene products in two human brain regions: frontal cortex and anterior cingulate gyrus (ACG), both with 10 cases and 10 controls. We first removed proteins with less than three samples in AD patients or normal controls, and then imputed missing values (0 or ‘#N/A’) by replacing them with averaged values from AD and control samples, separately. We constructed gene co-expression networks using the Pearson's correlation (*r* > 0.7) among AD patients and normal controls for each brain region. The co-expression networks were drawn using the ‘igraph’ (v1.2.4.1) R package.

#### Survival analysis

Among 342 AD risk genes, only 315 of them have expression data available. Among 315 AD risk genes, we carried out survival analysis (using the ‘survival’ R package) to identify ones that may predict by their expression levels the disease prognosis among AD patients. For each AD risk gene, using its expression profile (described above), we first separated AD patients into two groups, with either high (≥ the median) or low (< the median) expression of the gene. We then used the Kaplan–Meier estimator to compute estimated survivor functions, plotted them to directly compare survival between these two groups, and used the log-rank test to formally test whether their survival curves are identical.

### Prediction of AD risk variants

Risk variants contribute to the disease etiology and pathology mainly through two major mechanisms: coding variants may alter the function of gene products and non-coding variants may alter the transcription of genes by changing regulatory elements. Recently developed methods for variant annotation, such as LINSIGHT^13^, ExPecto^14^, and PrimateAI^15^, can quantify or provide useful information about the functional impact of genetic variants in the human genome, regardless of their potential connection to a specific disease or trait. By integrating such functional annotation of variants as a part of the computational framework that we developed for the post-GWAS analysis (Fig. 1), we are able to not only identify disease risk genes but also further predict disease risk variants. As LINSIGHT^13^, ExPecto^14^, and PrimateAI^15^ are so far the most completeness and up-to-date functional annotation of variants resources, we used them to prioritize the potential risk variant in this study. In this framework, we first use the variant type as the guide. For coding variants, we assume causality through the functional alteration of gene products and use their PrimateAI scores^15^, on a scale from 0 (benign) to 1 (pathogenic) measuring pathogenicity, to prioritize them. For non-coding variants, we assume causality is through gene regulation and prioritize variants in promoters by their ExPecto scores^14^, expression effect of fold change, and ones in enhancers by their LINSIGHT scores^13^, ranging from 0 to 1 and higher score indicating deleterious fitness consequences of the variants.

We systematically fine-mapped the genomic neighborhoods of predicted risk variants by analyzing all genotyped and imputed variants from the latest AD GWAS^5^. Only variants mapped to risk region and coding sequences and regulatory elements (i.e., promoters and enhancers) of AD risk genes were selected for downstream analysis. We used the following criteria to predict risk variants: PrimateAI scores, which measure the pathogenicity of coding variants from 0 (less pathogenic) to 1 (more pathogenic), are greater than 0.7; ExPecto scores, which predict functional impact of non-coding variants in promoters, are greater than ln(fold change = 1.2); and LINSIGHT scores, which measure the probability of negative selection on non-coding sites and can be used to prioritize SNVs associated with genetic diseases, are greater than 0.9.

### Transcription factor binding site motif analysis

The reference and the alternative alleles of each variant, along with ± 25 bp of flanking sequences, were analyzed using HOMER (v4.9.1)^98^. We used the findMotifs.pl program with the default parameters to find transcription factor binding site (TFBS) motifs in the reference and the alternative allele sequences, which were used as the background for each other to control the nucleotide context. A library of 364 vertebrate TFBS motifs in the format of position weight matrices was searched for matches. The matched motifs with scores greater than 7.5 were regarded as gain or loss of TFBS motifs.

## Supplementary Information

Supplementary Figures.

Supplementary Tables.

## Data Availability

Only publicly available data were used in this study. See the 'Data sets' subsection above for their availability. Computer code for major steps of data processing is available from the GitHub (https://github.com/zhenwang19/AD).
