# Supplementary material for: Deep post-GWAS analysis identifies potential risk genes and risk variants for Alzheimer’s disease, providing new insights into its disease mechanisms
Source: Sci Rep. 2021 Oct 15;11:20511. doi: 10.1038/s41598-021-99352-3 (PMC8519945; doi:10.1038/s41598-021-99352-3)
Supplement: Supplementary file 1 — Supplementary Figures. [file 41598_2021_99352_MOESM1_ESM.pdf]

Deep post-GWAS analysis identifies potential risk genes and risk variants for Alzheimer's disease, providing new insights into its disease mechanisms

Zhen Wang[1,2], Quanwei Zhang[2], Jhih-Rong Lin[2], M.Reza Jabalameli[2], Joydeep Mitra[2], Nha Nguyen[2], and Zhengdong Zhang[2,§]

[1] College of Animal Sciences, Zhejiang University, Hangzhou, Zhejiang, China.

[2] Department of Genetics, Albert Einstein College of Medicine, Bronx, New York, USA.

§ Corresponding author (E-mail: zhengdong.zhang@einstein.yu.edu)

SUPPLEMENTARY FIGURES

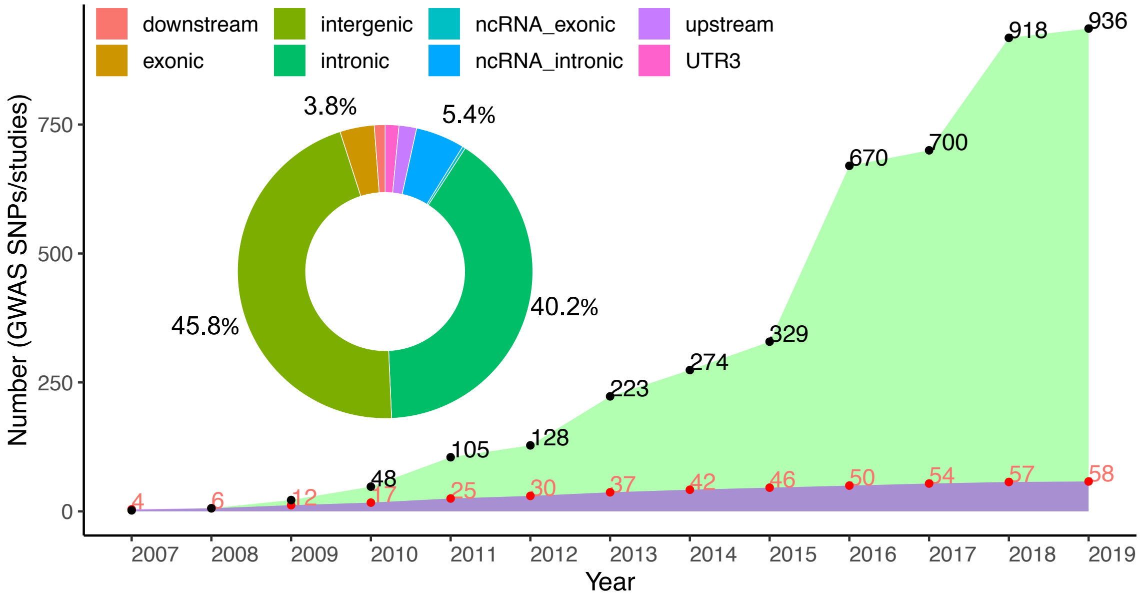

Supplementary Fig S1. GWAS of AD since 2007. The figure is based on data from the GWAS Catalog (1) (as of December 2018) and the latest GWAS of AD (2). The green area shows the total number of AD-associated SNPs, and the purple area shows the total number of GWAS of AD. The insert chart shows the proportions of different types of all 936 AD-associated SNPs.

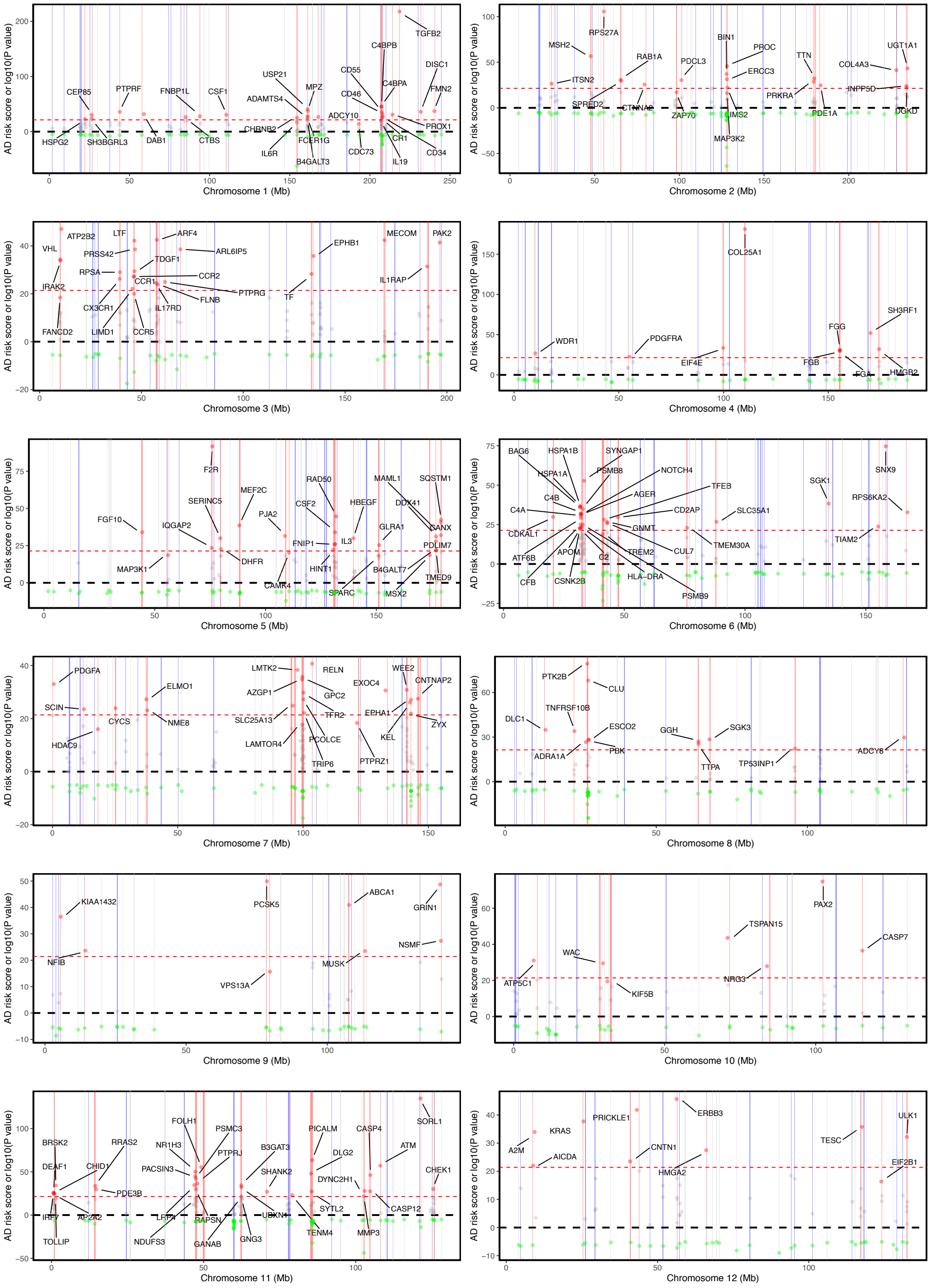
AD risk score or log10(P value)
Chromosome 1 (Mb)
TGFB2
C4BPB
DISC1
CD55
C4BPA
USP21
MPZ
FMN2
CEP85
PTPRF
FNBP1L
CSF1
CD46
ADAMTS4
ADCY10
PROX1
CHRNB2
FCER1G
CR1
HSPG2
SH3BGRL3
DAB1
CTBS
IL6R
CDC73
CD34
B4GALT3
IL19
Chromosome 2 (Mb)
RPS27A
MSH2
BIN1
UGT1A1
PROC
RAB1A
PDCL3
TTN
COL4A3
ITSN2
ERCC3
INPP5D
SPRED2
PRKRA
CTNNA2
ZAP70
IMS2
PDE1A
DGKD
MAP3K2
Chromosome 3 (Mb)
LTF
ARF4
MECOM
PAK2
ATP2B2
VHL
PRSS42
EPHB1
ARL6IP5
TDGF1
RPSA
CCR2
IRAK2
CCR1
PTPRG
IL1RAP
FLNB
TF
CX3CR1
IL17RD
FANCD2
LIMD1
CCR5
Chromosome 4 (Mb)
COL25A1
SH3RF1
FGG
WDR1
PDGFRA
EIF4E
FGB
FGA
HMGB2
Chromosome 5 (Mb)
F2R
RAD50
MAML1
SQSTM1
MEF2C
SERINC5
CSF2
HBEGF
DDX41
FGF10
PJA2
GLRA1
CANX
IQGAP2
FNIP1
IL3
PDLIM7
DHFR
HINT1
MAP3K1
B4GALT7
TMED9
CAMK4
SPARC
MSX2
Chromosome 6 (Mb)
BAG6
HSPA1B
SYNGAP1
HSPA1A
PSMB8
NOTCH4
SGK1
SNX9
C4B
AGER
TFEB
RPS6KA2
C4A
CD2AP
SLC35A1
CDKAL1
GNMT
TIAM2
TMEM30A
CUL7
ATF6B
APOM
C2
TREM2
HLA-DRA
CFB
CSNK2B
PSMB9
Chromosome 7 (Mb)
LMTK2
RELN
WEE2
PDGFA
EXOC4
CNTNAP2
ELMO1
AZGP1
GPC2
SCIN
TFR2
EPHA1
CYCS
SLC25A13
NME8
ZYX
KEL
HDAC9
PCOLCE
LAMTOR4
TRIP6
PTPRZ1
Chromosome 8 (Mb)
PTK2B
CLU
TNFRSF10B
DLC1
ESCO2
GGH
SGK3
ADRA1A
PBK
TTPA
TP53INP1
ADCY8
Chromosome 9 (Mb)
KIAA1432
PCSK5
ABCA1
GRIN1
NSMF
NFIB
MUSK
VPS13A
Chromosome 10 (Mb)
PAX2
TSPAN15
CASP7
WAC
ATP5C1
NRG3
KIF5B
Chromosome 11 (Mb)
SORL1
FOLH1
PSMC3
PICALM
CASP4
BRSK2
RRAS2
NR1H3
PTPRJ
B3GAT3
DLG2
ATM
DEAF1
CHID1
PACSIN3
SHANK2
DYNC2H1
CHEK1
PDE3B
SYTL2
CASP12
IRF7
AP2A2
LPXN
RAPSN
UBXN1
TOLLIP
NDUFS3
GANAB
GNG3
TENM4
MMP3
Chromosome 12 (Mb)
ERBB3
PRICKLE1
ULK1
KRAS
A2M
TESC
AICDA
CNTN1
EIF2B1
HMGA2

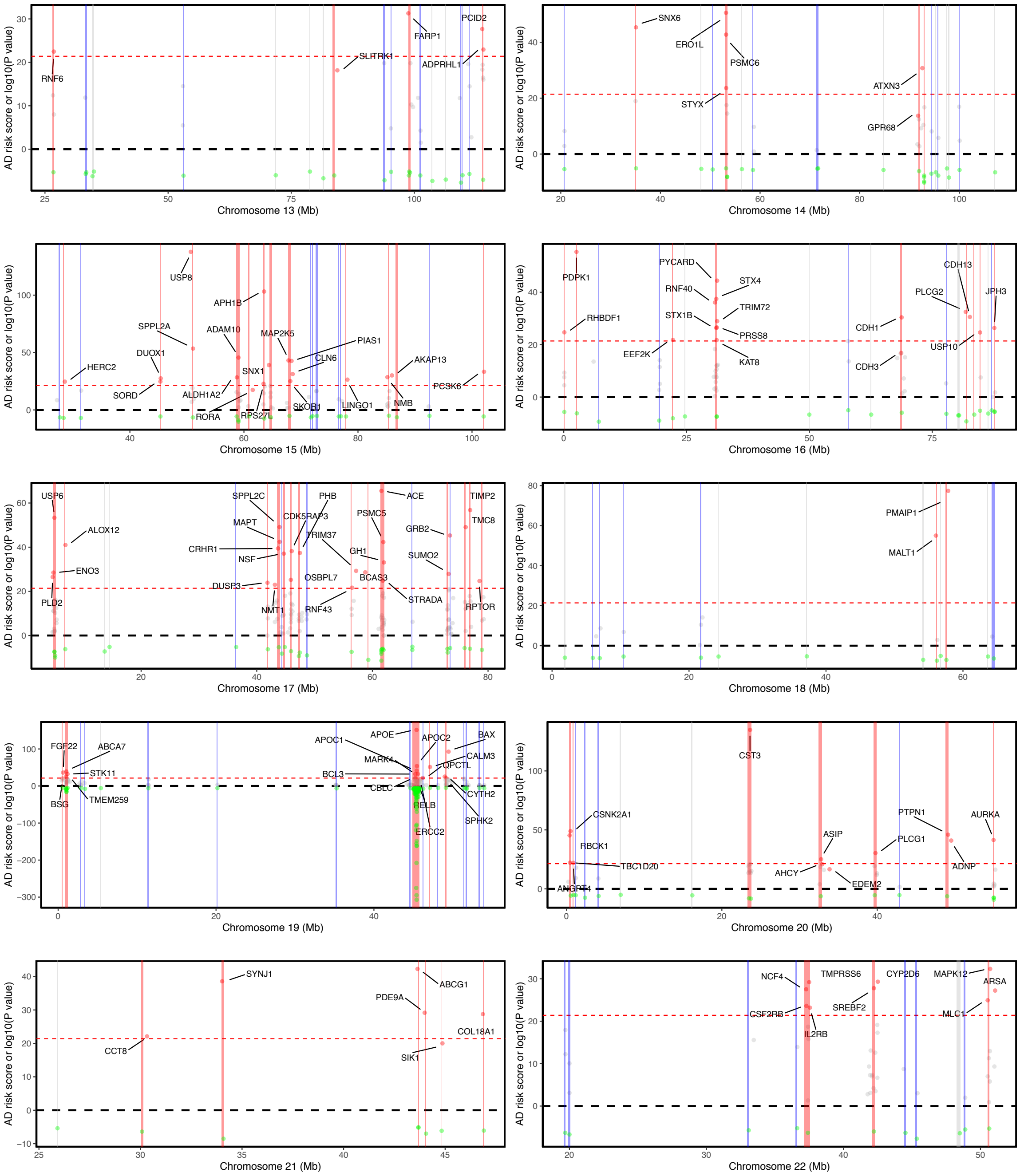

Supplementary Fig S2. GWAS AD SNPs, AD risk regions, AD risk gene candidates, and predicted AD risk genes in each human autosome. Green dots below the black dashed line represent GWAS AD SNPs and their $\log_{10}P$ values. Above the black dashed line, gray dots represent AD risk gene candidates, and red ones predicted AD risk genes (labelled with gene symbols). The red dashed line marks the threshold for AD risk gene prediction based on the stringent training. AD risk genes below this threshold were predicted with the lenient training gene set. Vertical lines represent AD risk regions: red for risk regions with AD risk genes, blue for risk regions with only AD risk gene candidates, gray for risk regions without any AD risk gene candidates.

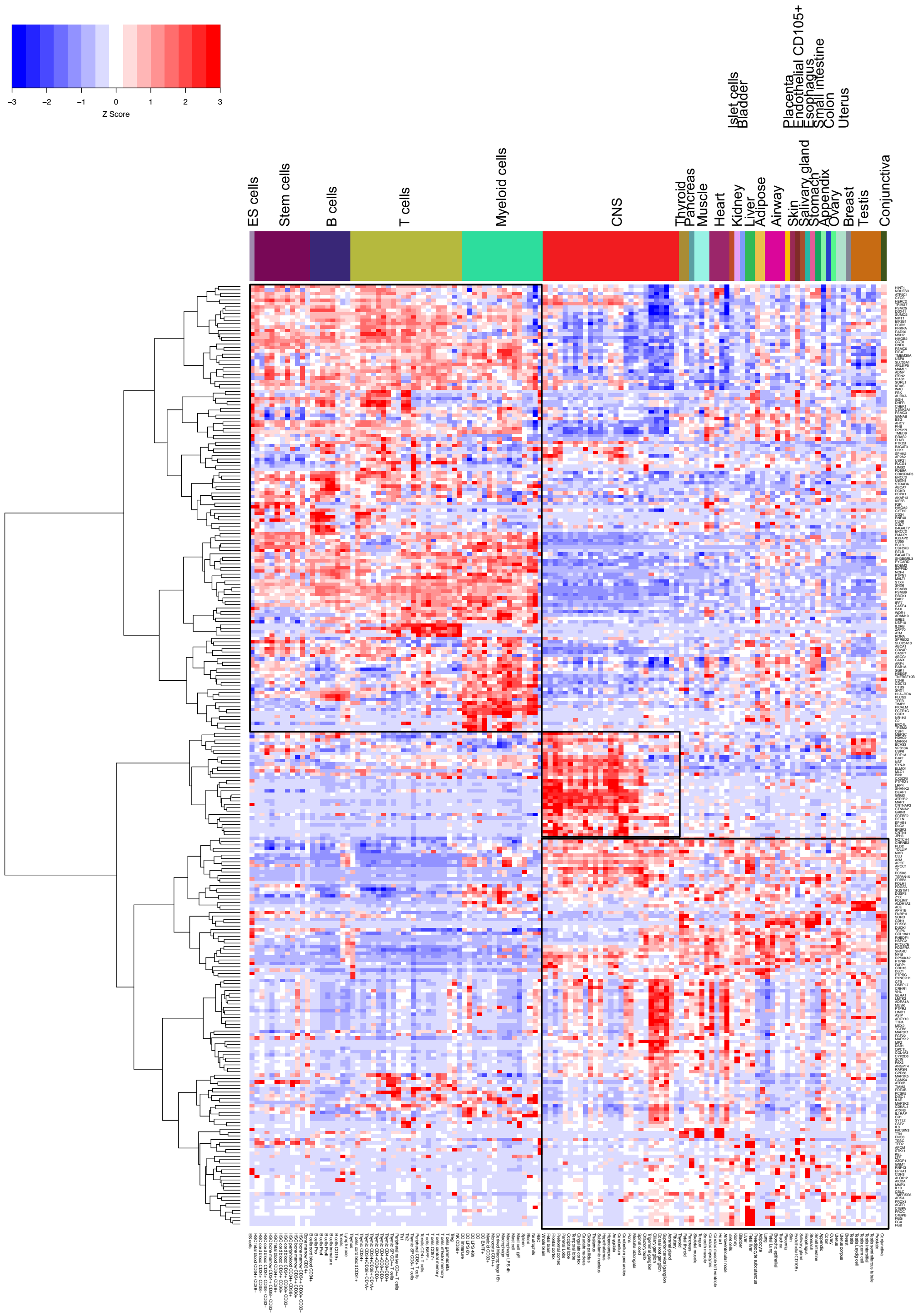

Supplementary Fig S3. Expression of AD risk genes in different tissues. Genes were clustered according to their expressions among different tissues. Three black boxes indicate gene clusters and corresponding tissues in which they are more transcriptionally active. 64 AD risk genes were not included due to the lack of data from the Gene Enrichment Profiler (http://xavierlab2.mgh.harvard.edu/EnrichmentProfiler/help.html).

The heatmap was plotted using R ‘gplots’ package (https://github.com/talgalili/gplots) with the ‘heatmap.2’ function.

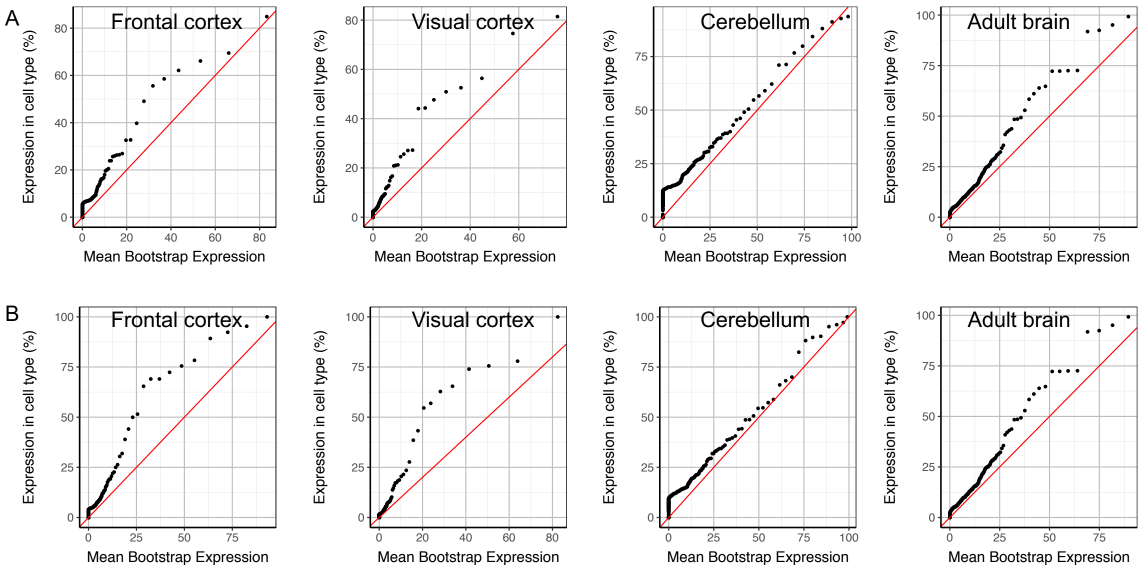

Supplementary Fig S4. Enriched expression of AD risk genes in endothelia and microglia from different brain regions. (A) Endothelia. (B) Microglia. The plot shows the actual level of expression of AD risk genes against the mean expression level of the *i*th most expressed gene in a bootstrapping analysis of the same number of genes. If the expression of AD risk genes was randomly distributed for a cell type from a brain region, the dots would be expected to fall along the red line. See Skene and Grant **(3)** for method details.

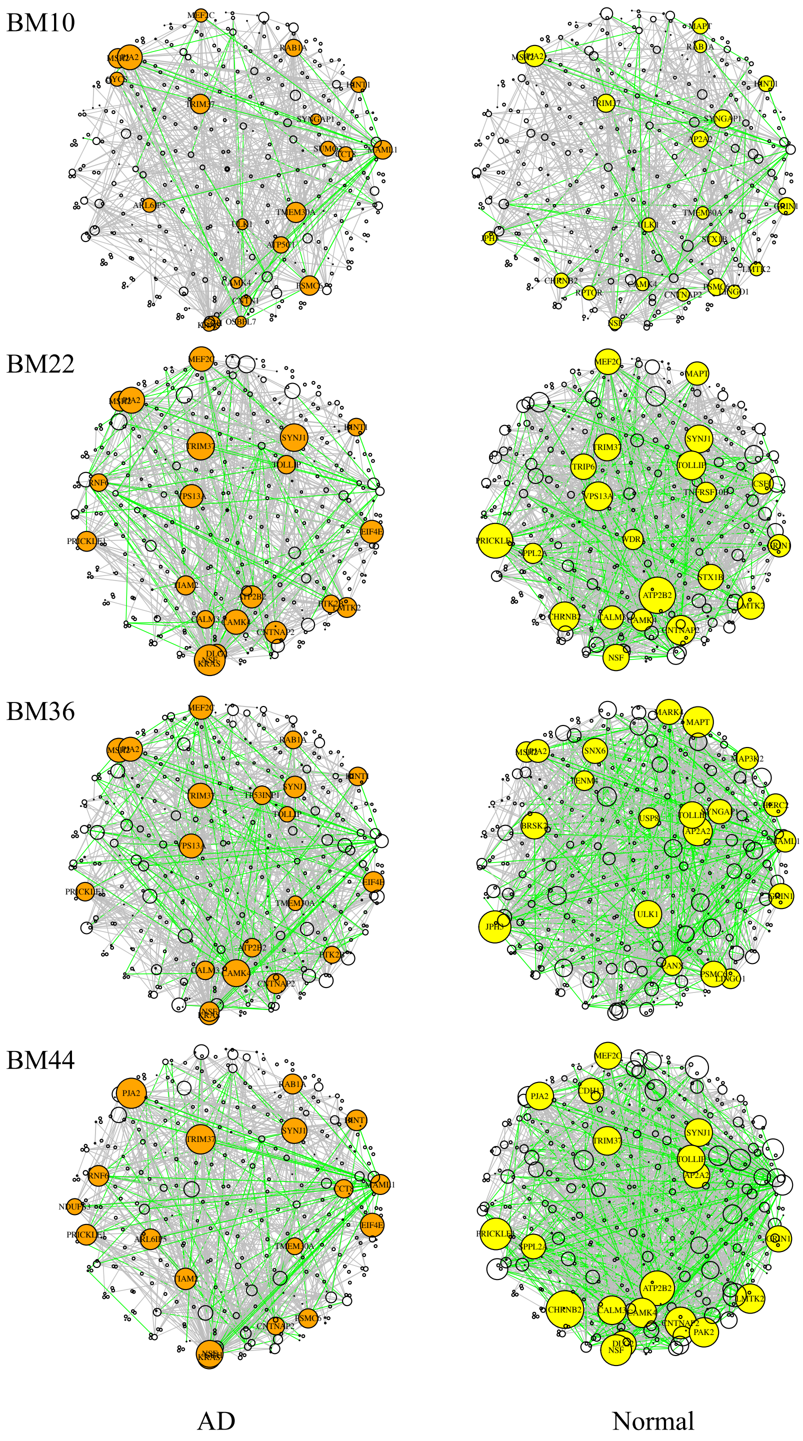

Supplementary Fig S5. Co-expression networks of AD risk genes in both AD patients and normal controls. Networks were constructed using the Pearson's correlation coefficients (> 0.7). Positive and negative correlations are denoted by gray and green lines, respectively. The size of a node is proportional to the connectivity of the gene. The network hub genes are colored orange or yellow.

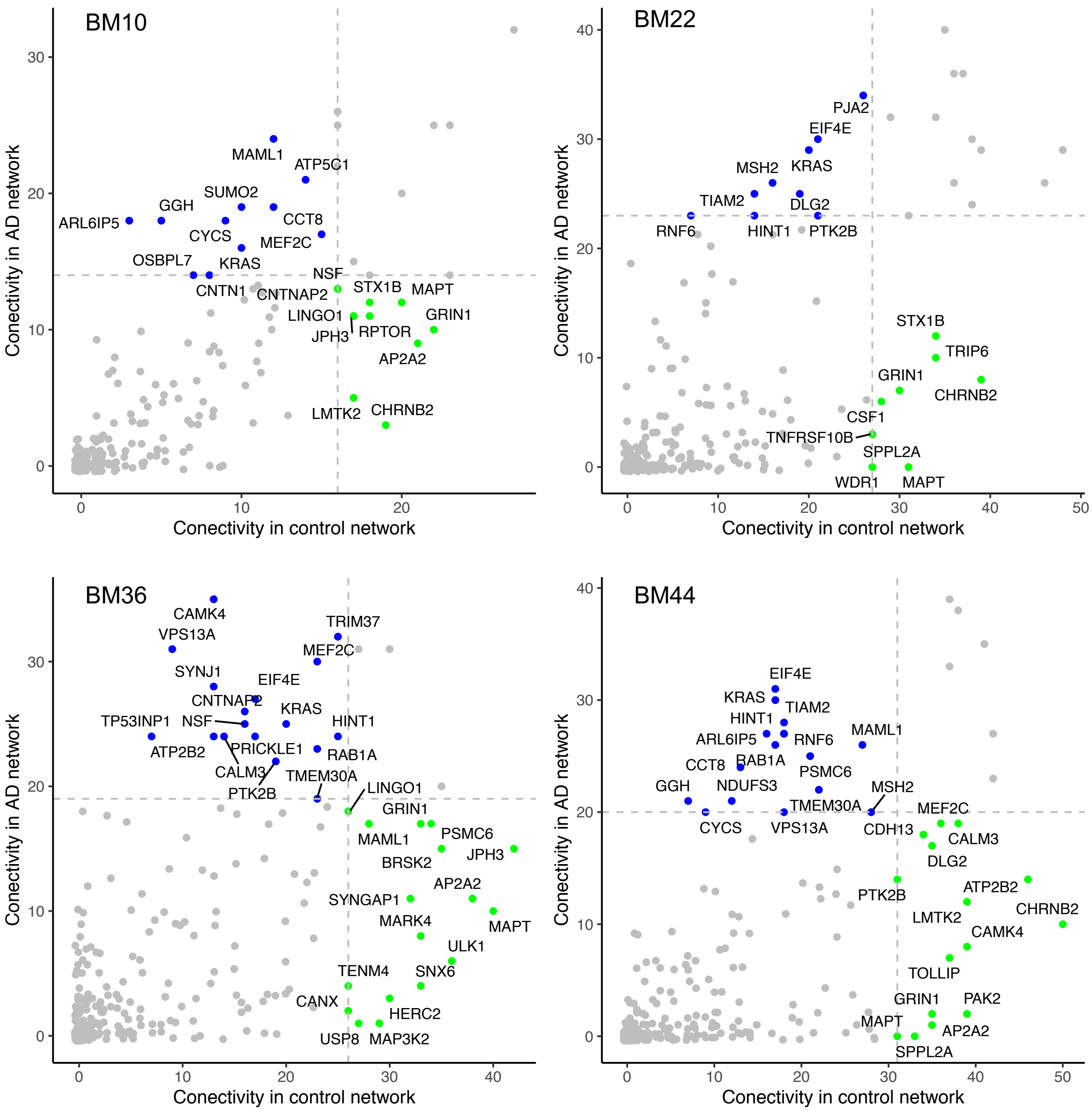

Supplementary Fig S6. Network connectivity of AD risk genes in co-expression networks of both AD patients and normal controls. Each dot represents an AD risk gene. The gray dashed line marks the threshold for network hub genes either in the co-expression network of AD patients or normal controls. Blue and green dots represent network hub genes only in the co-expression network of AD patients and only in the co-expression network of normal controls, respectively.

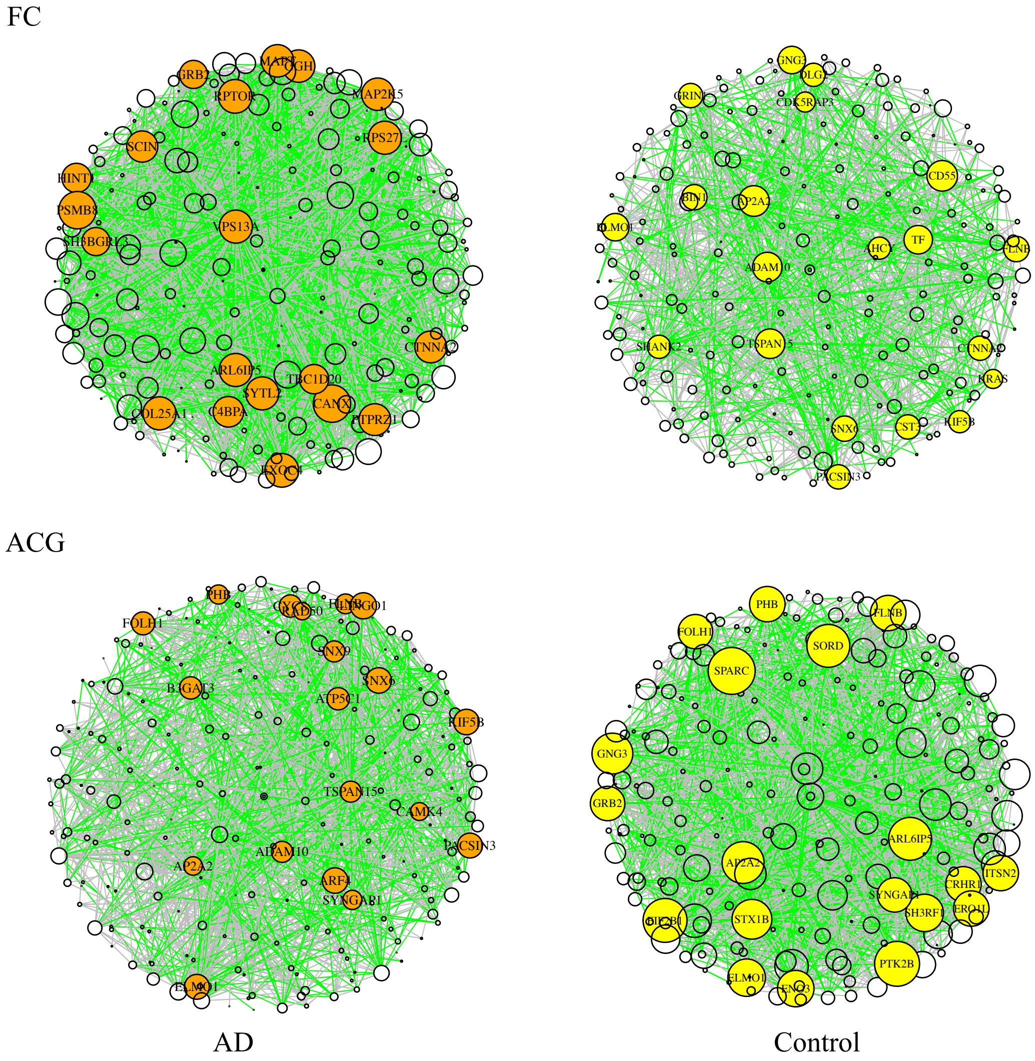

Supplementary Fig S7. Co-expression networks of AD risk genes products in both AD patients and normal controls. Co-expression networks were constructed using protein data and the Pearson's correlation coefficients (> 0.7). Positive and negative correlations are denoted by gray and green lines, respectively. The size of a node is proportional to the connectivity of the gene. The network hub genes are colored orange or yellow.

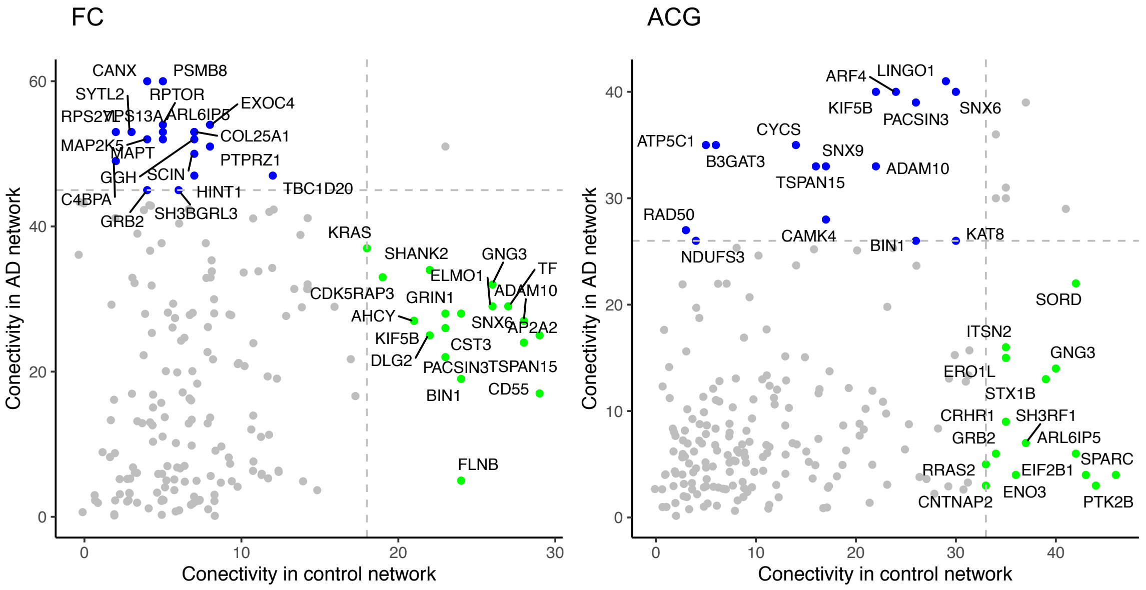

Supplementary Fig S8. Network connectivity of AD risk genes in protein-based co-expression networks of both AD patients and normal controls. Each dot represents an AD risk gene. The gray dashed line marks the threshold for network hub genes either in the co-expression network of AD patients or normal controls. Blue and green dots represent network hub genes only in the co-expression network of AD patients and only in the co-expression network of normal controls, respectively.

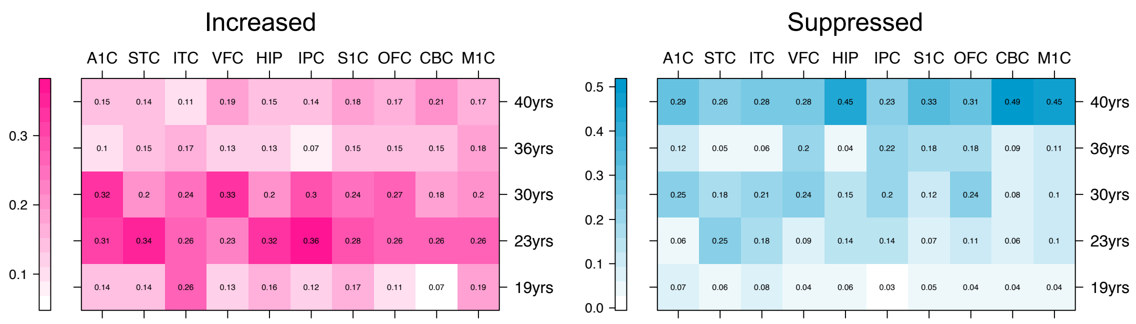

Supplementary Fig S9. Spatiotemporal expression patterns of AD risk genes during brain development. Both the active (red) and the suppressed (blue) gene expression were analyzed, using the same approach as in Lin et al. (4). Each heat maps shows gene expression at five developmental stages, in a chronological order, in ten brain regions. The shade of the color is proportional to the ratio of genes that manifest active (or suppressed) transcriptional activities, in the corresponding brain region and at the developmental stage, to the total number of AD risk genes. Abbreviations: A1C, primary auditory cortex (core); STC, posterior superior temporal cortex; ITC, inferior temporal cortex; VFC, ventrolateral prefrontal cortex; HIP, hippocampus; IPC, posteroventral (inferior) parietal cortex; S1C, primary somatosensory cortex (area S1, areas 3,1,2); OFC, orbitofrontal cortex; CBC, cerebellar cortex; M1C, primary motor cortex (area M1, area 4).

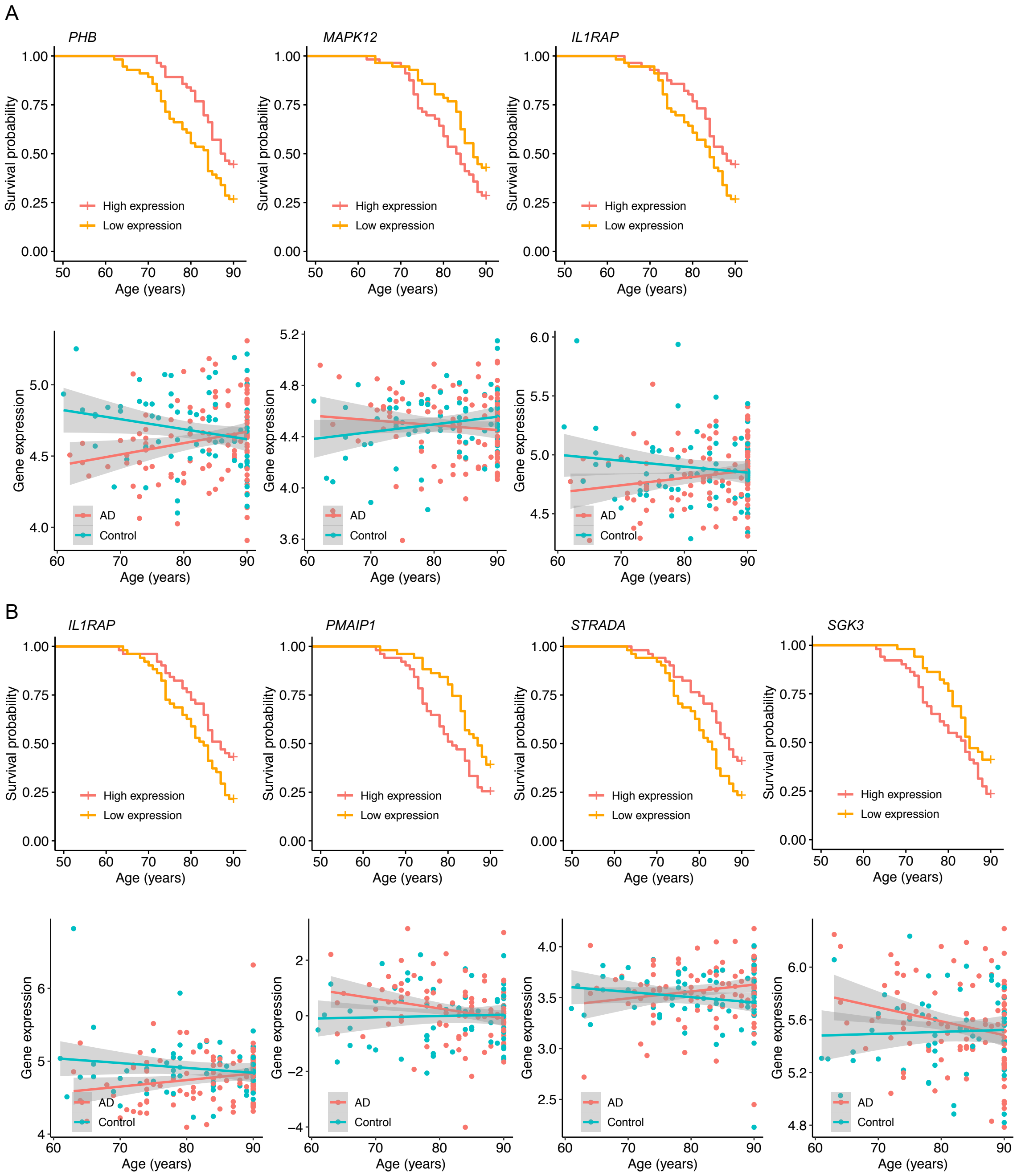

Supplementary Fig S10. AD survival curves and gene expression trajectories accompany with age. (A) BM10. (B) BM22. There are additional six genes, not shown in Fig 5, whose expression affects the survival of AD patients with low and high expression levels and shows opposite expression trajectories with age in AD patients and normal controls.

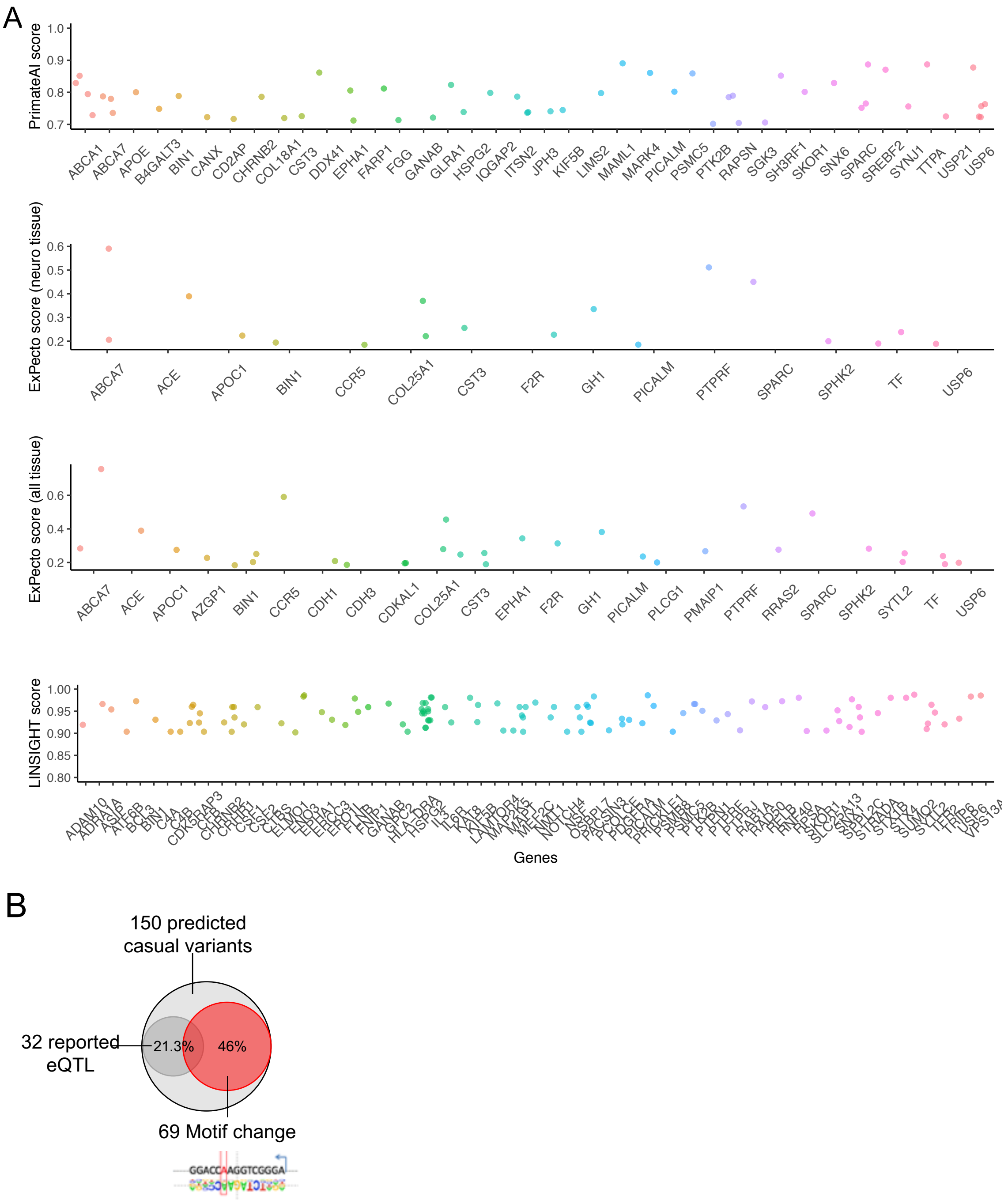

Supplementary Fig S11. Predicted AD risk variants. (A) AD risk genes, risk variants, and their functional scores. For promoter variants, we used ExPecto scores from both all or neuro tissues. (B) Functional annotation of predicted risk variants. ~57% (85/150) of them are either known eQTLs or predicted to change TFBS motifs.

REFERENCES

1. MacArthur, J., Bowler, E., Cerezo, M., Gil, L., Hall, P., Hastings, E., Junkins, H., McMahon, A., Milano, A., Morales, J. *et al*. (2017) The new NHGRI-EBI Catalog of published genome-wide association studies (GWAS Catalog). *Nucleic Acids Res*, **45**, D896-D901.
2. Jansen, I.E., Savage, J.E., Watanabe, K., Bryois, J., Williams, D.M., Steinberg, S., Sealock, J., Karlsson, I.K., Hagg, S., Athanasiu, L. *et al*. (2019) Genome-wide meta-analysis identifies new loci and functional pathways influencing Alzheimer's disease risk. *Nat Genet*.
3. Skene, N.G. and Grant, S.G. (2016) Identification of Vulnerable Cell Types in Major Brain Disorders Using Single Cell Transcriptomes and Expression Weighted Cell Type Enrichment. *Front Neurosci*, **10**, 16.
4. Lin, J.R., Cai, Y., Zhang, Q., Zhang, W., Nogales-Cadenas, R. and Zhang, Z.D. (2016) Integrated Post-GWAS Analysis Sheds New Light on the Disease Mechanisms of Schizophrenia. *Genetics*, **204**, 1587-1600.
